# Pharmacologic inhibition of Akt in combination with chemotherapeutic agents effectively induces apoptosis in ovarian and endometrial cancer cell lines

**DOI:** 10.1002/1878-0261.12888

**Published:** 2021-01-04

**Authors:** François Fabi, Pascal Adam, Sophie Parent, Laurence Tardif, Monique Cadrin, Eric Asselin

**Affiliations:** ^1^ Department of Medical Biology Université du Québec à Trois‐Rivières Canada

**Keywords:** AZD5363, capivasertib, chemoresistance, doxorubicin, endometrial cancer, ovarian cancer

## Abstract

The PI3K/Akt signaling pathway, the most frequently altered signaling system in human cancer, is a crucial inducer of dysregulated proliferation and neoplastic processes; however, few therapeutic strategies using PI3K/Akt inhibitors singly have been shown to be effective. The purpose of this paper was to underline the potential benefit of pharmacological modulation of the PI3K/Akt pathway when combined with specific chemotherapeutic regimens. We have studied the ability of NVP‐BEZ235 (PI3K/mTOR inhibitor) and AZD5363 (Akt inhibitor) in the sensitization of cancer cells to cisplatin and doxorubicin. Our results show that NVP‐BEZ235 sensitizes cells preferentially to cisplatin while AZD5363 sensitizes cells to doxorubicin. At equal concentrations (5 μm), both inhibitors reduce ribosomal protein S6 phosphorylation, but AZD5363 is more effective in reducing GSK3β phosphorylation as well as S6 phosphorylation. Additionally, AZD5363 is capable of inducing FOXO1 and p53 nuclear localization and reduces BAD phosphorylation, which is generally increased by cisplatin and doxorubicin. Finally, the combination of AZD5363 and doxorubicin induces apoptosis in cells and robustly reduces cell ability to clonally replicate, which underlines a potential cooperative effect of the studied compounds.

## Graphical abstract

The use of Akt inhibitor AZD5363 sensitized ovarian and endometrial cancer cells to the chemotherapeutic drug doxorubicin. A combination of AZD5363 and doxorubicin activated pro‐apoptotic pathways, namely through the inhibition of BAD phosphorylation as well as through the nuclear localization of FOXO1 and p53. This collaborative effect appears to be a potential tool to overcome chemoresistance in these neoplastic entities.

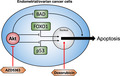

**Abbreviations**
BAD: BCL2‐associated agonist of cell death
CCD: charge‐coupled device
FOXO1: forkhead box protein O1
GSK3B: glycogen synthase kinase 3
MAPK: mitogen‐activated protein kinase
mTOR: mechanistic target of rapamycin
MTT: 3‐[4,5‐dimethylthiazole‐2‐yl]‐2,5‐diphenyltetrazolium bromide
p53: tumor protein P53
p70S6K: Ribosomal protein S6 kinase beta‐1
PBS/T: phosphate‐buffered saline/Tween
PI3K: phosphoinositide 3‐kinase
PTEN: phosphatase and tensin homolog
S6: ribosomal protein S6
XIAP: X‐linked inhibitor of apoptosis protein

## 1 Introduction

The PI3K(phosphoinositide 3‐kinase)/Akt signaling pathway is one of the most fundamental regulators of cellular processes and holds a central place in the integration of cell fate decisions. In that context, it is not surprising that the PI3K/Akt network is the most frequently altered signaling system in human cancers and that mutations in their associated genes are some of the most prevalent driver mutations when comparing all cancer types [1, 2]. Novel studies using powerful and refined methods of analysis have also shown that PI3K, along with Akt1 and the mTOR (mechanistic target of rapamycin) signaling pathway, is significantly more involved in cancer development than any other genes. The authors also posit, and demonstrate, that mutations should not be construed as the strongest predictor of molecular role in oncogenesis but rather that protein overexpression, and thus presumably enhanced activation and signaling, is the most impactful event in tumor development [3]. Taken together, these data underline the crucial role of PI3K/Akt signaling axis in the molecular dysregulations that underpins tumorigenesis and its impact on the ability of the cells to evade their own endogenous, orderly system of homeostatic dynamics. In accordance with this hypothesis, the PI3K/Akt/mTOR axis has been shown to be of the utmost importance in the capability of tumor cell to resist apoptosis induced by various therapies; this role is particularly well recognized in the context of gynecological malignancies [4, 5, 6, 7, 8, 9, 10, 11]. Some of the more common therapeutic strategies involve platinum‐based compounds or anthracyclines. More specifically, cisplatin is one of the most well described and used chemotherapeutic compound for the treatment of ovarian and endometrial cancer; doxorubicin, on the other hand, is often used in combination to cisplatin in ovarian cancer, in the event of a platinum‐refractory ovarian neoplasia, and in the context of high stage endometrial cancers [12, 13, 14]. Ovarian cancer and endometrial cancer are both diseases that exhibit high level of chemoresistance that is linked to overactivation of Akt, in some cases directly influenced by PTEN (phosphatase and tensin homolog) loss, in response to chemotherapeutic insults [4, 6, 9, 15]. When discovered at early stages, such as when the tumor is still restricted to the true pelvis, cytoreductive therapy accompanied by adjuvant chemotherapy will often allow complete remission [16, 17]. Unfortunately, ovarian cancer is generally diagnosed at later stages, characterized by widespread peritoneal and omental metastases which vastly reduces the effectiveness of surgery. In these cases, chemotherapy will also be used but will almost inevitably be thwarted by ovarian cancer's chemoresistant nature, thought to mainly derive from its extensive spatiotemporal tumor heterogeneity [18]. On the other hand, while a large portion of endometrial cancers initially respond positively to chemotherapy, the majority of recurrent endometrial malignancies will not display such sensitivity [19, 20, 21, 22, 23, 24, 25, 26]. One distinct exception should be mentioned, which is platinum‐sensitive recurrent ovarian cancer, a disease entity that is not fully understood but that is characterized by platinum sensitivity in tumors with a PFI (platinum‐free interval) of 6 months or more [27]. It is therefore necessary that we develop novel‐targeted therapeutic strategies that enhance chemosensitivity of these tumor types and restore treatment effectiveness; the overwhelming representation of mutations involving abnormal activation of the PI3K/Akt/mTOR axis also provides a strong support for the use of pharmacological tools suppressing the functions of this molecular pathway.

Accordingly, multiple strategies can be used to inhibit the activation and signaling events that unfold upon PI3K stimulation. While the earliest molecules, such as LY294002 and Wortmannin, acted in an upstream fashion by directly inhibiting PI3Ks, they were limited in their use because of their poor pharmacodynamics and low tolerability [28, 29, 30]. However, their ability to interfere with the PI3K/Akt/mTOR pathway and their effectiveness *in vitro* underlined the robust potential of this molecular strategy in cancer therapy. Novel inhibitors of PI3K were thus developed, exhibiting powerful pharmacological capabilities, high bioavailability, and advantageous dosability in patients. One such compound is NVP‐BEZ235, an orally bioavailable dual PI3K/mTOR inhibitor capable of selectively abrogating class I PI3K as well as mTOR kinase [31]. On the other hand, AZD5363 has been developed to act directly on all three isoforms of Akt, downstream of PI3K [32]. This molecule, rather than acting as an allosteric inhibitor of the Akt kinases, acts as a potent ATP‐competitive kinase domain inhibitor, preventing Akt phosphotransferase activity. In that context, while NVP‐BEZ235 inhibition of PI3K results in reduced Akt phosphorylation in line with other compounds of this class [31], AZD5363 inhibition of Akt has been shown to induce its hyperphosphorylation accompanied by a robust reduction in its downstream signaling capabilities [32].

A recent Cochrane review on PI3K/mTOR/Akt inhibitors also underlines that very few studies have explored the specific role of Akt inhibition, rather focusing on mTOR and PI3K blockades [33]. It is also interesting to note that many trials using PI3K or Akt inhibitors as a single intervention, or without the use of cytotoxic chemotherapy, in the context of gynecological cancers (NCT01226316; [34], NCT01307631; [35], NCT00920257; [36], NCT01283035; [37]) showed at best moderate to absent effectiveness, even in the context of cancers presenting mutations in the PI3K/Akt/PTEN/Ras axis. On the other hand, it should be noted that when combined with other compounds, PI3K/Akt inhibitions are generally given with taxanes or platinum‐based agents (NCT02476955; results pending, NCT01653912; [38], NCT00431054; [39], NCT04561817; results pending), with variable effectiveness. To our knowledge, no investigation regarding the specific combination of AZD5363 (Capivasertib) with doxorubicin has been conducted.

In that context, we endeavored to measure whether NVP‐BEZ‐235 or AZD5363 could preferentially potentiate the pro‐apoptotic ability of cisplatin and doxorubicin through the inhibition of the PI3K/Akt/mTOR axis of signaling. Using multiple gynecological cancer cell lines as models, we first evaluated the ability of each drug to modulate key signaling pathways. We then combined both kinase inhibitors with both chemotherapeutic agents to establish whether cooperative effects could be observed. After identifying the favorable combination of NVP‐BEZ235 with cisplatin and AZD5363 with doxorubicin, we further characterized the molecular events explaining this enhanced effectiveness. Our results suggest that AZD5363, when combined with doxorubicin, allows a cooperative sensitization of cancer cells to the latter molecule through the inhibition of Akt and mTOR signaling pathways, downregulation of key inhibitors of apoptosis as well as nuclear localization of tumor suppressors such as FOXO1 (forkhead box protein O1) and p53 (tumor protein P53). This highlights the potential clinical interest of the combined use of an Akt inhibitor such as AZD5363 with a cytocidal compound, more specifically with doxorubicin, in the context of gynecological malignancies.

## 2 Methods

### 2.1 Cell lines and reagents

OVCAR‐3, HEC‐1A, and SKOV‐3 cell lines were purchased from ATCC (Manassas, VA, USA). A2780 and A2780CP were kindly provided by G. Peter Raaphorst (Ottawa Regional Cancer Center, Ottawa, Canada). Ishikawa cells were kindly provided by S. Mader (Université de Montréal, Montréal, Canada). ECC‐1 cells were kindly provided by N. Gévry (Université de Sherbrooke, Sherbrooke, Canada). EN‐1078D were previously isolated from a poorly differentiated stage IIIC endometrial adenocarcinoma presenting ovarian and ganglionic metastasis [40]. All cell lines were maintained in temperature‐controlled incubator at 37 °C and at 5% CO_2_. All culture media were supplemented with gentamicin with a final concentration of 50 mg·L^−1^. Specific culture conditions were as follows: OVCAR‐3: RPMI‐1640 media supplemented with 10% FBS. Hec‐1A: McCoy's 5A‐modified media supplemented with 5% BGS. SKOV‐3: McCoy's 5A‐modified media supplemented with 10% FBS. A2780, 2780CP, and Ishikawa: DMEM/F12 media supplemented with 2% BGS. ECC‐1: RPMI‐1640 media supplemented with 10% FBS. EN‐1078D: DMEM/F12 media supplemented with 10% BGS.

All the antibodies were obtained from Cell Signaling Technology (Danvers, MA, USA) with the exception of the anti‐rabbit secondary antibody used for western blotting (Bio‐Rad Laboratories, Hercules, CA, USA) and for the Alexa Fluor 488‐tagged anti‐rabbit secondary antibody, which was obtained from Thermo Fisher Scientific Inc. (Waltham, MA, USA). NVP‐BEZ235, AZD5363, cisplatin, and doxorubicin were obtained from SelleckChem (Houston, TX, USA). AZD5363 and NVP‐BEZ235 were obtained from SelleckChem. AZD5363 was diluted in DMSO at a stock concentration of 10 mm; NVP‐BEZ235 was diluted in DMSO at a stock concentration of 2 mm; cisplatin was diluted in physiological saline at a stock concentration of 3.33 mm; doxorubicin was diluted in H_2_O at a stock concentration of 10 mm. In all experiments, control conditions recapitulated the highest working concentration of diluents used in the treatment conditions of that experiment, in order for the diluent's effect on cell viability to be taken into account.

### 2.2 MTT assays

Briefly, plates were seeded with 180 μL of cells in suspension (for Ishikawa, 16 000; ECC‐1, 14 000; A2780/CP, 16 000; HEC‐1A, 12 000; SKOV‐3, 12 000) in medium using 96‐well plates. Plates were incubated at 37 °C, 5% CO_2_ for 24 h. Cisplatin, doxorubicin, AZD5363, and NVP‐BEZ235 were diluted in fresh medium, serially diluted, and added to the plates to obtain the final indicated concentrations. Cell were then incubated for another 24 h after which 10 μL of 3‐(4,5‐dimethylthiazol‐2‐yl)‐2,5‐diphenyltetrazolium bromide (MTT) [5 mg·mL^−1^ in PBS (phosphate‐buffered saline)] was added to the wells. Four hours later, 100 μL of the solubilization solution [10% sodium dodecyl sulfate (SDS) in 0.01 m HCl] was added and the plates incubated overnight (37 °C, 5% CO_2_). The optical density was read using a FLUOstar Optima BMG (BMG Labtech Inc., Durham, NC, USA) at 565 nm. Each experiment was performed in duplicate on the same plate. The results shown are representative of three independent experiments.

### 2.3 Western blot analysis

After the end of the treatment period, both floating and attached cells were collected and cell lysate was done using cold radioimmunoprecipitation assay lysis buffer containing protease inhibitors (Complete; Roche Applied Science, Indianapolis, IN, USA), followed by three freeze–thaw cycles. Proteins were measured using the Bio‐Rad DC protein assay. Western blotting was performed as previously described [41]. Appropriate peroxidase‐conjugated secondary antibodies were used, and the blot was developed using SuperSignal West Femto substrate (Thermo Scientific, Rockford, IL, USA), as described by the manufacturer, using a cooled CCD (charge‐coupled device) camera (UVP System). The shown results are representative of at least three independent experiments.

### 2.4 Colony formation assays

Cells were plated at a confluence of 2000 cells per well in a 6‐well plate and grown for 24 h. Cells were then treated for 24 h after which the media was replaced. Cells were allowed to grow for 10 days, and media was replaced every 5 days. After 10 days, cells were washed with PBS and fixed in ice‐cold formalin for 10 min. After fixation, colonies were colored with Giemsa Stain 0.4% for 5 min. Plates were then washed with running water and allowed to dry, and colonies were photographed using a cooled CCD camera. Images were quantified using the colonyarea software [42].

### 2.5 Immunofluorescence

Cells were treated as described above and were grown in 6‐well plates containing sterile coverslips. On the day of analysis, cells were fixed with 4% paraformaldehyde for 10 min and permeabilized for 10 min using 0.1% Triton X‐100 in 0.1% sodium citrate at room temperature. After blocking with 4% normal goat serum blocking for 1 h, cells were incubated with a primary antibody at a concentration of 1 μg·mL^−1^ or an isotypic control antibody for 1 h. After incubation, cells on the coverslips were washed three times with PBS and then incubated with Alexa Fluor 488 secondary antibodies (1 : 800 dilution) for 30 min at room temperature in dark conditions. Cells were counterstained with Hoechst 33248 (0.25 μg·mL^−1^) for 5 min, and slides were mounted using SlowFade Gold Antifading reagent (Invitrogen, Carlsbad, CA, USA) and viewed under a Leica TCS SP8 confocal microscope, using a 63× immersion lens (Leica Microsystems, Concord, ON, Canada).

### 2.6 Statistical analyses

Statistical analysis was done by one‐way analysis of variance with Tukey's *post hoc* test or two‐way analysis of variance followed by multiple comparison tests when appropriate. Statistical significance was accepted when *P* < 0.05. *P < 0.05; **P < 0.01; ***P < 0.001. All analyses were performed using graphpad prism software, version 8 (GraphPad Software, Inc., La Jolla, CA, USA).

## 3 Results

### 3.1 NVP‐BEZ235 and AZD5363 are capable of interfering with the PI3K/Akt/mTOR axis in ovarian and endometrial cancer cell lines

We first studied the ability of AZD5363 and NVP‐BEZ235 to inhibit various proteins that act as markers of PI3K/Akt/mTOR activation. Completely upstream of that signaling pathway, we measured the complete activation of Akt through phosphorylation on serine 473 [2]; we also measured GSK3β (glycogen synthase kinase 3 beta) phosphorylation on serine 9, a direct target of activated Akt, and an effective measure of GSK3β inhibition that reduces its ability to act as a tumor suppressor [43]. On the other hand, we measured phosphorylation of p70S6K (Ribosomal protein S6 kinase) on Thr389, a direct marker of growth factor influence on mTOR axis activation and a critical residue post‐translational modification necessary for p70S6K activation that could potentially act as an indicator of therapy effectiveness [44, 45]; consequently, we also measured S6 protein phosphorylation on serine 235/236, a direct substrate of p70S6K (p70) of which the phosphorylation allows 5′TOP‐dependent translation and a potential marker of chemoresistance [46, 47]. Figure S1 shows MTT data pertaining to cell line sensitivity to both chemotherapeutic compounds and inhibitors when treated singly.

**Fig. 1:**
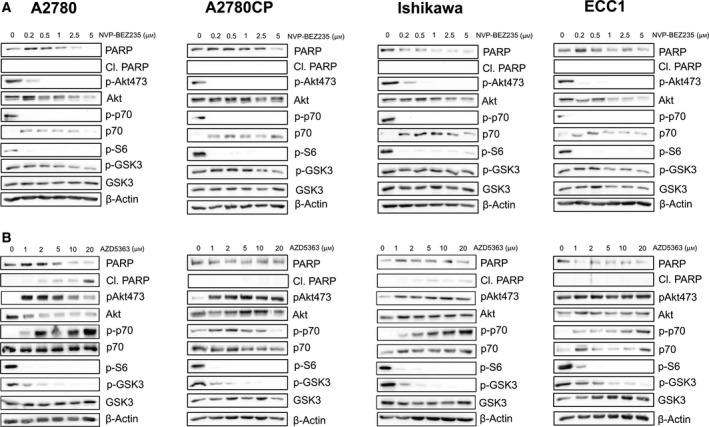
NVP‐BEZ235 and AZD5363 modulate signaling pathways in a dose‐dependent manner. (A) Cell lines were treated with increasing concentration of NVP‐BEZ235 (0–5 μm). Western blot was performed using relevant antibodies, and β‐actin was used as a loading control. Results shown are representative of three independent experiments. (B) Cell lines were treated with increasing concentration of AZD5363 (0–20 μm). Western blot was performed using relevant antibodies, and β‐actin was used as a loading control. Results shown are representative of three independent experiments. [Colour figure can be viewed at wileyonlinelibrary.com]

We thus treated A2780, an ovarian chemosensitive cell line, A2780CP, an allogenic cell line derived from A2780 that is highly resistant to both doxorubicin and cisplatin, Ishikawa, an endometrial cisplatin sensitive, doxorubicin‐resistant cell line and finally ECC‐1, an endometrial cisplatin and doxorubicin‐resistant cell line, with increasing doses of NVP‐BEZ235 or AZD5363 (Fig. 1A). Results show that NVP‐BEZ235 is capable, at low doses, of fully inhibiting Akt phosphorylation on serine 473 in all tested cell lines while not inducing apoptosis, as shown by the absence of PARP cleavage. Incidentally, p70 phosphorylation was also reduced in correlation with the loss of Akt phosphorylation, as was the phosphorylation of p70 prime substrate, S6. Interestingly, NVP‐BEZ235 seemed to be incapable of fully abrogating S6 phosphorylation in Ishikawa cell line, even at high dose. Finally, NVP‐BEZ235 was unable to impede GSK3β phosphorylation at all doses, underlining the inability of the drug to completely abrogate GSK3β inactivation by Akt. AZD5363, on the other hand, seemingly heightened Akt phosphorylation on serine 473, an effect that is cogent with its pharmacological activity as an ATP‐competitive inhibitor [32, 48]. Additionally, AZD5363 at high doses induced apoptotic cell death in A2780 and Ishikawa cell lines. Interestingly, AZD5363 seemed to increase the phosphorylation of p70, which could be due to the high homology of the ATP pocket present between Akt and p70 [32]. Nonetheless, S6 phosphorylation was completely abrogated even at low doses, underlining the ability of AZD5363 to interfere with mTOR axis activation. Finally, the compound was very effective in reducing GSK3β phosphorylation.

### 3.2 NVP‐BEZ235 and AZD5363 can enhance the ability of cisplatin and doxorubicin of reducing cancer cell viability

**Fig. 2:**
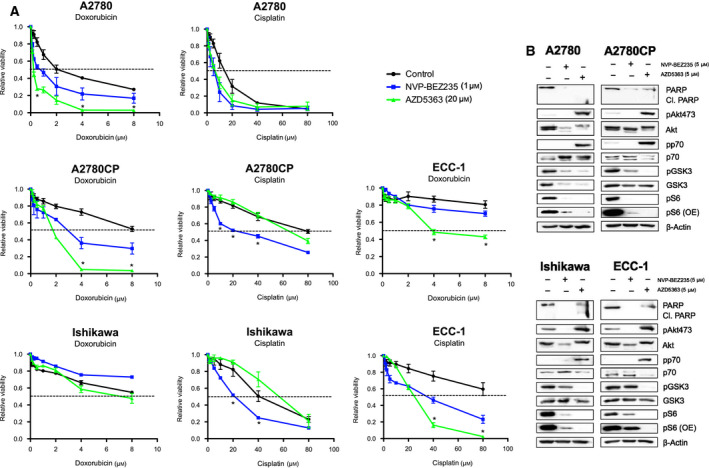
Combination of AZD5363 or NVP‐BEZ235 sensitizes cells to cisplatin and doxorubicin. (A) Cell lines were treated with increasing concentration of cisplatin (0–80 μm) or doxorubicin (0–8 μm) in the presence or absence of either AZD5363 (20 μm) or NVP‐BEZ‐235 (1 μm) for 24 h. MTT assays were then used to determine changes in cell viability. (B) Cell lines were treated with equal concentration of NVP‐BEZ235 or AZD5363 (5 μm). Western blot was performed using relevant antibodies, and β‐actin was used as a loading control. One‐way ANOVA followed by Tukey's *post hoc* test was performed. All data are means ± SEM of three independent experiments. *P < 0.05. [Colour figure can be viewed at wileyonlinelibrary.com]

The data obtained in Section 3.1 suggested that the studied compounds could modulate the PI3K/Akt/mTOR axis. We thus sought to determine whether the combination of either NVP‐BEZ235 or AZD5363 with cisplatin or doxorubicin could potentiate the effects of these cytocidal molecules in the context of cancer cell lines. We treated cells with fixed doses of inhibitors concomitantly with increasing doses of chemotherapeutic drugs for 24 h. Concentration of 20 μm of AZD5363 was used in cellular models with robust effects [48, 49]; results found in Fig. 1 showed that 20 μm was effective in reducing pathway activations in all cell lines. It has also been reported that doses exceeding 10 μm are easily achievable at tolerable *in vivo* dosages, highlighting the pharmacodynamic capabilities of the compound [50]. In parallel, our previous experiments, in addition to studies performed by other groups, had suggested that a NVP‐BEZ235 at a dose of 1 μm was effective when combined with cisplatin and was capable, by itself, of inhibiting multiple PI3K‐dependent pathways [51, 52, 53, 54]. The results obtained in Fig. 1 confirmed that this dose could potently inhibit signaling pathways but was mostly incapable of inducing apoptosis in tested cell lines. We performed MTT assays to determine the ability of each combination to achieve some form of cooperation in reducing cell viability (Fig. 2A). The results showed that in A2780, AZD5363 was significantly more effective than NVP‐BEZ235 in sensitizing cells to doxorubicin treatments. This was also the case in A2780CP cells, where AZD5363 sensitized cells to doxorubicin more effectively than NVP‐BEZ235; however, the latter was significantly more effective in sensitizing A2780CP cells to cisplatin. In the case of Ishikawa cell lines, no significant differences were found in the case of doxorubicin while NVP‐BEZ235 allowed sensitization to cisplatin treatments. Finally, in ECC‐1, both doxorubicin and cisplatin sensitivity were enhanced in cells treated with AZD5363. We also wanted to determine the relative effectiveness of both inhibitors when compared directly to each other. To evaluate their respective effectiveness at similar dosages, cells were treated with AZD5363 or NVP‐BEZ235 at 5 μm and western blots were performed (Fig. 2B). The results show that, altogether, AZD5363 is clearly more effective in reducing S6 phosphorylation and GSK3β phosphorylation, two important downstream targets of the PI3K/Akt/mTOR axis. Taken together, these results suggest that the AZD5363 preferentially potentiates the action of doxorubicin while NVP‐BEZ235 presents a similar effect with cisplatin; however, AZD5363 appears to more powerfully impede Akt/mTOR activity.

### 3.3 AZD5363 potentiates the activity of doxorubicin and allows the induction of apoptosis

**Fig. 3:**
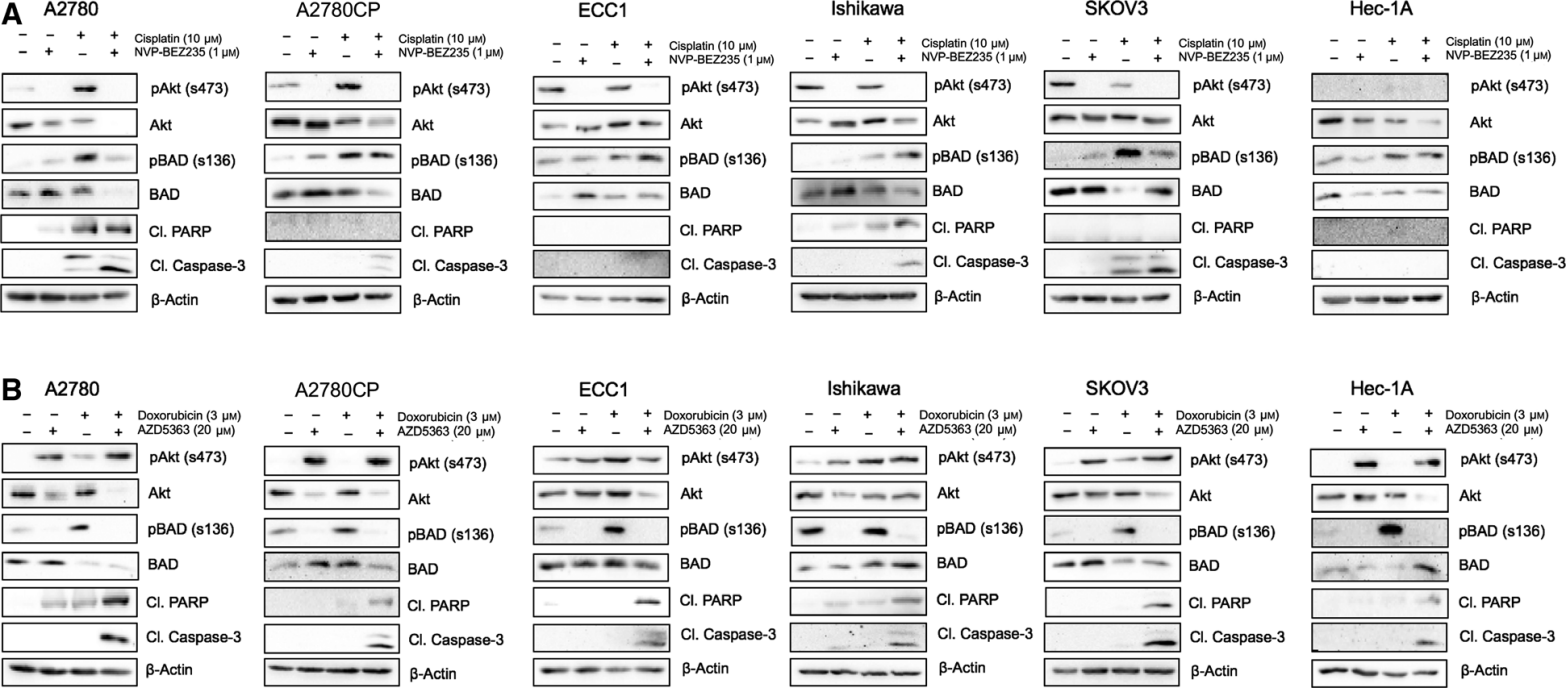
Combination of AZD5363 with doxorubicin induces apoptosis in gynecological cancer cell lines. (A) Cell lines were treated with either cisplatin (10 μm), NVP‐BEZ‐235 (1 μm), or a combination of both for 24 h. Western blot was performed using relevant antibodies, and β‐actin was used as a loading control. (B) Cell lines were treated with either doxorubicin (3 μm), AZD5363 (20 μm), or a combination of both for 24 h. Western blot was performed using relevant antibodies, and β‐actin was used as a loading control. Results shown are representative of three independent experiments. [Colour figure can be viewed at wileyonlinelibrary.com]

Considering the parameters suggested by the results obtained in Section 3.2, we combined treatments of doxorubicin (3 μm) with AZD5363 (20 μm), a dose that is incapable of inducing apoptosis in resistant cell lines when used singly, and observe the obtained cooperative effects. Accordingly, considering the seemingly more effective combination of NVP‐BEZ235 with cisplatin, cells were treated in parallel with cisplatin (10 μm) and NVP‐BEZ235 (1 μm). Cells were again treated for 24 h with either drugs or a combination of both. Multiple cell lines were used to ascertain the validity and applicability of our findings; briefly, SKOV‐3 is an aggressive, high‐grade ovarian cancer cells resistant to cisplatin and doxorubicin while Hec‐1A cells are medium grade endometrial cells resistant to cisplatin and doxorubicin [55]. The results of the combination of NVP‐BEZ235 with cisplatin suggest that the two compounds can act cooperatively in A2780CP cells and Ishikawa cells, as shown by the appearance of caspase‐3 cleavage only in the context of concomitant treatment; however, PARP cleavage was unobservable in either, underlining the incomplete process of apoptosis that seems to occur. Interestingly, NVP‐BEZ235 failed, either singly or in combination with cisplatin, to reduce BAD (BCL2‐associated agonist of cell death) phosphorylation, which could explain the lack of effectiveness observed using this therapeutic strategy (Fig. 3A). On the other hand, AZD5363 combination with doxorubicin allowed robust caspase‐3 cleavage in all cell lines, accompanied by PARP cleavage. Additionally, while most cells responded to doxorubicin treatment with a sharp increase in BAD phosphorylation, AZD5363 treatment completely abrogated this effect, which could partly explain the excellent effectiveness of this drug combination (Fig. 3B).

### 3.4 Combination of AZD5363 and doxorubicin regulates key modulators of the apoptotic responses as well as the translocalization of tumor suppressors

**Fig. 4:**
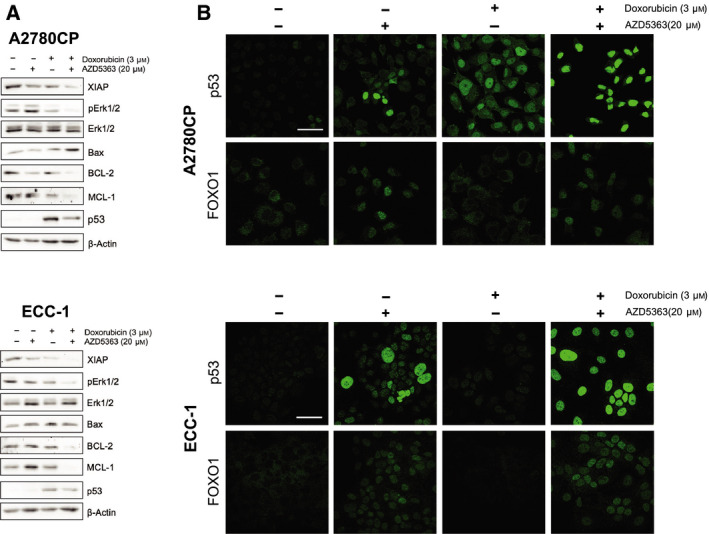
Combination of AZD5363 with doxorubicin regulates key pro‐apoptotic signaling pathways. (A) Cell lines were treated with either doxorubicin (3 μm), AZD5363 (20 μm), or a combination of both for 24 h. Western blot was performed using relevant antibodies, and β‐actin was used as a loading control. Results shown are representative of three independent experiments. (B) Immunofluorescence experiments were conducted in order to determine the effect of the previous treatments on p53 and FOXO1 subcellular localization. Scale bar = 45 μm. [Colour figure can be viewed at wileyonlinelibrary.com]

In order to characterize more effectively the effect of AZD5363 combination with doxorubicin, we elected to conduct further experiments involving A2780CP and ECC‐1 cell lines, as they are both good representation of respectively ovarian and endometrial cell lines presenting a robust resistance profile to chemotherapy [56, 57, 58, 59]. Again, cells were submitted to 24 h of treatments using doses of 3 μm for doxorubicin and 20 μm AZD5363 and additional western blot experiments were performed (Fig. 4A). Doxorubicin seemed to allow p53 stabilization in both cell lines, an effect that was still observable when the treatment was combined with AZD5363. In both cell lines, the combination therapy proved capable of reducing XIAP (X‐linked inhibitor of apoptosis protein) expression, as well as robustly reducing Erk activation. Interestingly, A2780CP displayed increased Bax and reduced BCL‐2, while the same treatment induced only a downregulation of BCL‐2 in ECC‐1 cells. Finally, in both cases, the concomitant treatment of the cells with both an inhibitor and a cytotoxic compound allowed an almost complete abrogation of MCL‐1 protein levels. We then sought to determine the effect of the treatments on two well‐characterized transcription factors acting as tumor suppressors, FOXO1 and p53 (Fig. 4B). In both cell lines, FOXO1 is completely absent in the nuclear compartment in the control setting; this is reversed upon single therapy using AZD5363, which allows nuclear localization and protein stabilization. A similar effect can be observed with p53, which is present at low level in the nuclear, as well as cytoplasmic, compartment; however, treatment with AZD5363 allows nuclear accumulation of the protein. In the case of FOXO1, doxorubicin has no noticeable effects on protein localization; p53, however, localizes to the nucleus in A2780CP cells upon doxorubicin treatment, an effect that is not apparent in ECC‐1. Finally, the combined treatment allows maximal nuclear localization of both p53 and FOXO1 in A2780CP as well as ECC‐1.

### 3.5 Combination of AZD5363 and doxorubicin significantly decreases tumor cells ability to replicate clonally

**Fig. 5:**
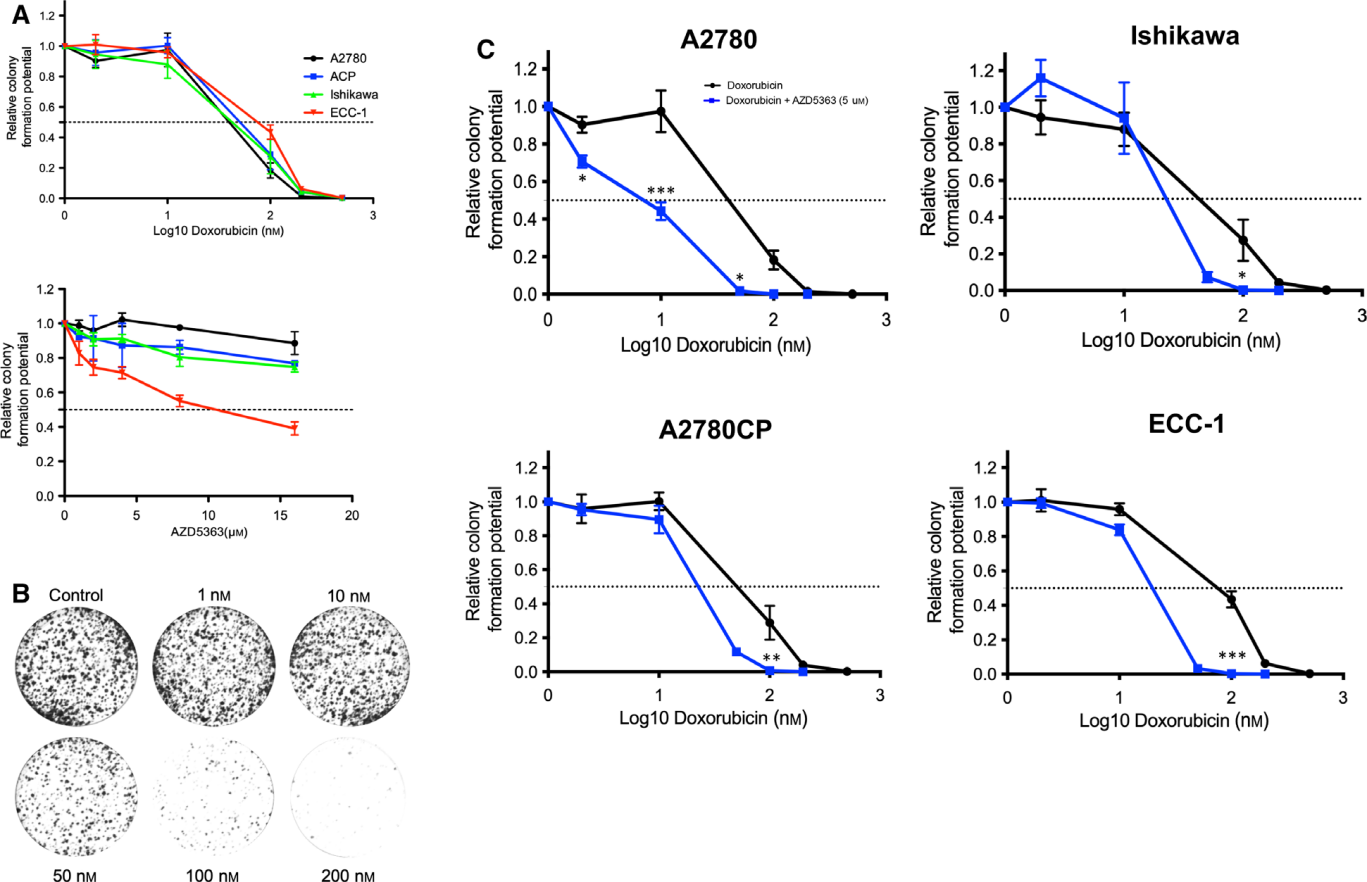
AZD5363 potentiates the ability of doxorubicin to reduce cell proliferation and clonal replication. (A) Cell lines were treated with increasing concentrations of AZD5363 (0–16 μm) or doxorubicin (0–500 nm). (B) Representative clonogenic assay; in this instance, all cells were pretreated with AZD5363 (5 μm) and with increasing concentrations of doxorubicin (0–200 nm). Densitometric analysis was conducted using colonyarea software. (C) Cell lines were treated with increasing concentrations of doxorubicin (0–200 nm) in the presence of AZD5363 (5 μm). Densitometric analysis was conducted using colonyarea software. Two‐way ANOVA followed by Tukey's *post hoc* test was performed. All data are means ± SEM of three independent experiments. *P < 0.05, **P < 0.01, ***P < 0.001. Corresponding calculated IC50 can be found in Table 1. [Colour figure can be viewed at wileyonlinelibrary.com]

In order to inquire the long‐term effect of the cooperation between AZD5363 and doxorubicin on cell viability and induction of apoptosis, we performed clonogenic assays, which allows a more translatable measure of the effect of the combined treatment on tumor progression. Additionally, we believe that clonogenic assays grant us the ability to measure with more validity the effect of longer term, lower concentration treatments on studied cancer cell population, as well as emulate the selection of a cellular subpopulation and its subsequent clonal expansion, features that are coherent with the clinical use of chemotherapy. Preliminary experiments suggested that AZD5363, when used singly, was incapable of reducing cell viability by 50% or more, except in the case of ECC‐1 where the IC50 was found to be approximately 10 μm. By performing nonlinear regressions, we measured the IC50 of doxorubicin in all four cell lines; the obtained results are recapitulated in Table 1 (Fig. 5A). Unsurprisingly, ECC‐1 and A2780CP cell lines were most resistant to doxorubicin when used singly. We then combined increasing doses of doxorubicin with 5 μm of AZD5363, a dose that we had shown in Sections 3.1 and 3.2 to be effective in abrogating multiple signaling pathways as well as being incapable, as seen in Fig. 5A, to reach IC50. The obtained results clearly demonstrate the capabilities of AZD5363 in sensitizing resistant cell lines to doxorubicin, as shown by the stark change displayed in dose–response curves as well as IC50, which can be found in Table 1 (Fig. 5C). Overall, our results suggest that AZD5363, when used in combination with doxorubicin, sensitizes cancer cell lines to the latter compound cytotoxic effect, which translates into the inhibition of cell clonal duplication abilities.

**Table 1:** Doxorubicin IC50 obtained through nonlinear regressions.

	A2780	A2780CP	Ishikawa	ECC‐1
Monotherapy (Doxorubicin) (nm)	52.15	67.85	55.75	95.53
Combined therapy (AZD5363 + Doxorubicin) (nm)	6.07	29.09	26.03	22.3

## 4 Discussion

The resistance of gynecological cancer to cytotoxic agent is one of the most critical challenges in the treatment of these diseases. Many demonstration points toward the idea that particular pathways, acting as cornerstone of cellular physiology and nonwithstanding the presence or absence of mutated nodes within them, could be determinant in the emergence of cancer hallmark characteristics. We believe that the PI3K/Akt/mTOR pathway, due to its almost singularly central nature, is of the utmost importance in regulating the phenomenon of chemoresistance. It has been well demonstrated that this axis of signaling is critical in cancer establishment, progression, and sustainment. In this paper, we have endeavored to examine whether the combination of a PI3K/mTOR inhibitor, NVP‐BEZ235, or an Akt ATP‐competitive inhibitor, AZD5363, could sensitize cancer cells to the cytocidal activity of cisplatin or doxorubicin. Altogether, the results we obtained suggest that AZD5363 strongly sensitizes cancer cell lines to the effect of doxorubicin, even in the context of chemoresistant cell lines.

Interestingly, as shown by Fig. 4A, p53 is enhanced by single‐agent therapy involving doxorubicin, possibly through intrinsic pathway activation and p53 stabilization; however, maximal nuclear localization can be seen in the context of concomitant therapy, as shown in Fig. 4B. Therefore, we are allowed to think that doxorubicin, by itself, is capable of inducing p53 expression and possible stabilization, which has been reported by other research groups in different cellular contexts [60]; AZD5363, on the other hand, potentiates its action by allowing maximal nuclear localization and potentially relieving molecular hindrances limiting p53 activity. The combination of AZD5363 and doxorubicin was particularly effective in modulating tumor‐suppressing transcription factor translocalization and potential activation. Considering that p53 and FOXO1 could potentiate each other transcriptional activity, the concomitant nuclear localization of these two transcription factors could be critical to their respective function and allows the observed drug's cooperative effects [61, 62]. This experiment demonstrates very simply the idea that single‐agent therapy could elicit the cellular response, in that case transcription factor nuclear translocation, while being unable to induce apoptosis. The combination of these inhibitors, allowing adequate signaling pathways response, must thus be paired with an apoptotic insult of sufficient potency in order to obtain the desired antitumoral effect.

One of the main differences that can be observed between both inhibitor compounds is the inability of NVP‐BEZ235 to impede GSK3β phosphorylation. Indeed, our results suggest that AZD5363 was more effective in impeding the Akt/mTOR pathway activation than NVP‐BEZ235, considering their similar effects at various doses with the added AZD5363 capability of robustly reducing GSK3β phosphorylation. Considering the ambiguous role of GSK3β activity in cancer, the fact that AZD5363 allows sensitization to doxorubicin while relieving GSK3β of inhibitory post‐translational modification allows us to surmise that, in our context, this kinase acts as a tumor suppressor. Experiments conducted in multiple models have shown that GSK3β can stabilize the β‐catenin complex, thus acting as a promoter of dysregulated cell differentiation; GSK3β could potentially even act as an enabler of chemoresistance, and its overexpression has been reported as a driver of drug resistance in some ovarian cancer samples [63, 64]. It is plausible that multiple axis of regulation impacts GSK3β in our models; as such, more work is necessary to fully decipher the canvas of post‐translational modifications affecting this complex kinase, allowing its oscillation between tumor suppressor and oncogene. Alternatively, it is also well recognized that inhibitors of upstream pathways, such as PI3K inhibitors, generally allow cross talk compensation with various signaling networks, mainly the MAPK/Erk (mitogen‐activated protein kinase) pathway [65, 66]. We are allowed to think, considering the cardinal necessity of cancer cells to regulate their homeostatic capabilities, that the inhibition of one of the most central pathways allowing their survival and proliferation would provoke the activation of parallel pathways in order to prevent cell suicide. In that case, the use of PI3K inhibitors would have the unwanted effect of stimulating alternate pathways, an effect that would be mitigated in the case of AZD5363, which disables solely Akt signaling, possibly preventing unwanted balancing effects. Also, the failure of NVP‐BEZ235 to inhibit BAD phosphorylation, one of the most striking effects of AZD5363, could explain the higher efficacy of the latter in inducing apoptosis, especially considering that abnormal Akt activation, and thus substrate phosphorylation, is one of the most well‐recognized molecular process of chemoresistance [2, 4, 67, 68]. Alternatively, as seen in Fig. 3A, some level of cleaved caspase‐3 can be observed without an increase in PARP cleavage; it is highly plausible that the combination of NVP‐BEZ235 and cisplatin is capable of activating the intrinsic pathway of apoptosis but fails to reduce downstream inhibitors of apoptosis such as XIAP, which could explain this discrepancy.

Of course, the nature of the mechanisms allowing a seemingly preferentially effective combination of NVP‐BEZ235 with cisplatin and AZD5363 with doxorubicin will require further experiments to fully elucidate. The underlying mechanism of action of doxorubicin, which does not solely damage DNA through intercalation but also disrupts topoisomerase, could partially explain this difference [69]. Further experiments are required to fully characterize the molecular events driving the potentially synergistical tumoricidal effects that we have described. There is also the distinct possibility that AZD5363, being an Akt inhibitor in addition to a potential inhibitor of p70S6K, prevents the concomitant activation of parallel, mTOR‐dependent signaling pathways which usually follows Akt inhibition. This specific characteristic of AZD5363 could impede the dysregulated compensation mechanisms from which chemoresistance often emerge. However, the inhibitory activity of AZD5363 could be partially lost if cisplatin is capable of inducing the activation of these same pathways, an effect that could be mitigated by doxorubicin different mechanism of action and distinct resistance mechanisms [70]. Finally, while some data have shown that PI3K/Akt inhibitors generally display enhanced effectiveness in the case of PTEN‐ and PI3K‐mutated cell lines, our results suggest that the combination of such an inhibitor with a well‐suited cytotoxic compound could alleviate these limitations; further experiments will have to be conducted in order to characterize more completely the parameters that underpin the effectiveness of this approach.

## 5 Conclusions

Overall, we believe that the administration of Akt inhibitors such as AZD5363 allows the robust sensitization of gynecological cancer cell lines to doxorubicin by removing molecular roadblocks that would impede the induction of apoptosis; this effect, however, might not be limited to cells originating from these tissues. This combinatorial therapeutic strategy, which allows the upregulation of crucial pro‐apoptotic proteins with the coordinated downregulation of anti‐apoptotic regulators, could be used to potentiate the treatments currently used in the clinic. Most importantly, it bears to remind that most investigations have focused on taxanes or platinum‐based compound combination with PI3K/Akt inhibitors. To conclude, our results suggest that PI3K and Akt blockades are preferentially and asymmetrically effective with specific compounds that are often considered similar because of their cytocidal mechanism of action. We also report that combination of AZD5363 with doxorubicin appears superior to the combination of NVP‐BEZ235 with cisplatin. Taken together, we believe further studies involving the combination of Akt inhibitors with doxorubicin could potentially yield positive results and eventually lead to a novel chemotherapeutic regimen.

## Conflict of interest

The authors declare no conflict of interest.

## Author contributions

FF, SP, and EA conceived and designed the experiments. FF, PA, and LT performed the experiments. FF analyzed the data. EA and MC contributed reagents/materials/analysis tools. FF and EA wrote the paper. All authors read and approved the final manuscript.

### Peer Review

The peer review history for this article is available at https://publons.com/publon/10.1002/1878‐0261.12888.

## Supporting information


**Fig. S1.** Effects of single agent therapy on gynecological cancer cell lines A. Cell lines were treated with increasing concentration of cisplatin (0‐80μM), doxorubicin (0‐8μM), AZD5363(0‐40μM) or NVP‐BEZ‐235(0‐4μM) for 24h. MTT assays were then used to determine changes in cell viability. All data are means ± SEM of three independent experiments.

## Data Availability

We will gladly share any data, materials, and protocols used in the experiments reported.
