# Supplementary figures and images for: Pharmacologic inhibition of Akt in combination with chemotherapeutic agents effectively induces apoptosis in ovarian and endometrial cancer cell lines
Source: Mol Oncol. 2021 Jan 4;15(8):2106–19. doi: 10.1002/1878-0261.12888 (PMC8334290; doi:10.1002/1878-0261.12888)

Supp. Fig 1

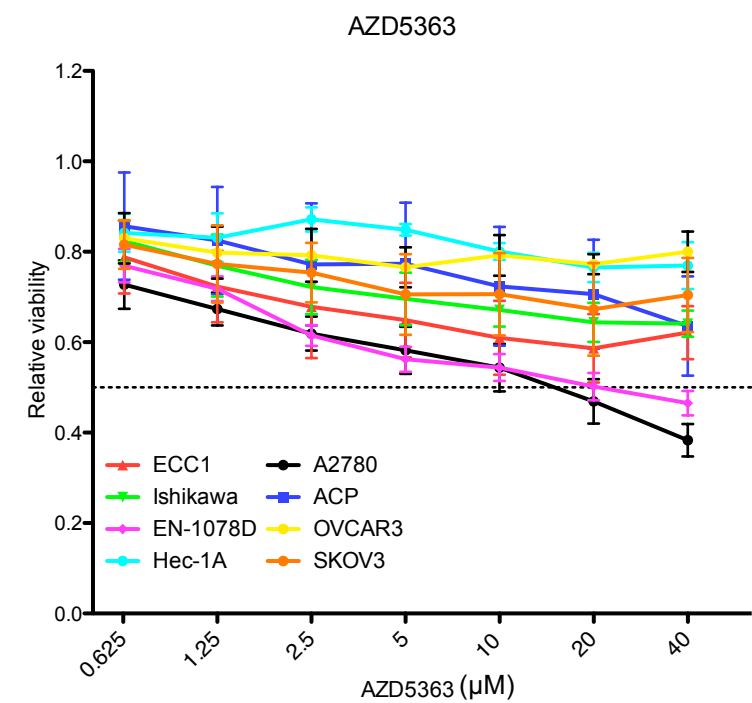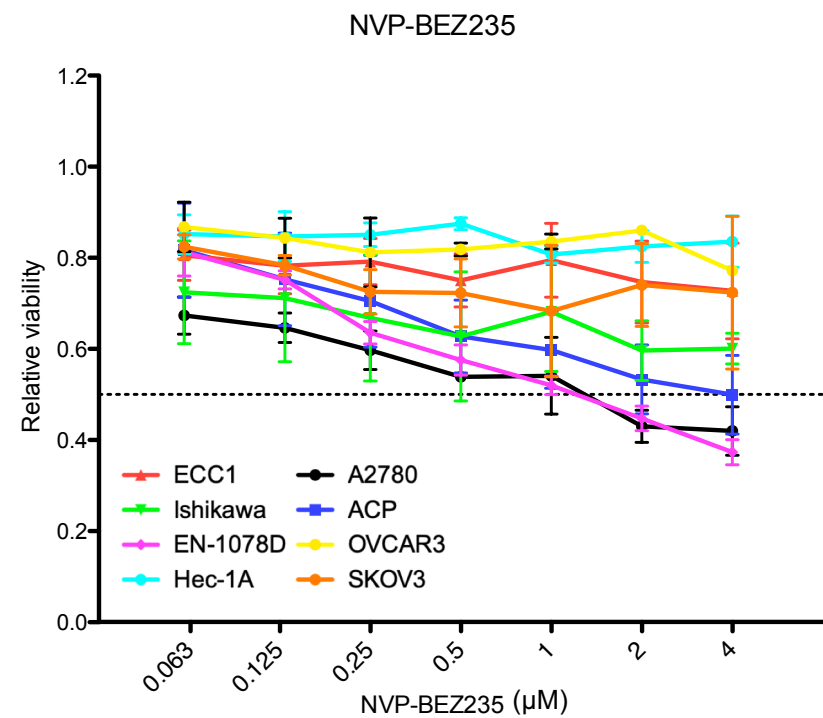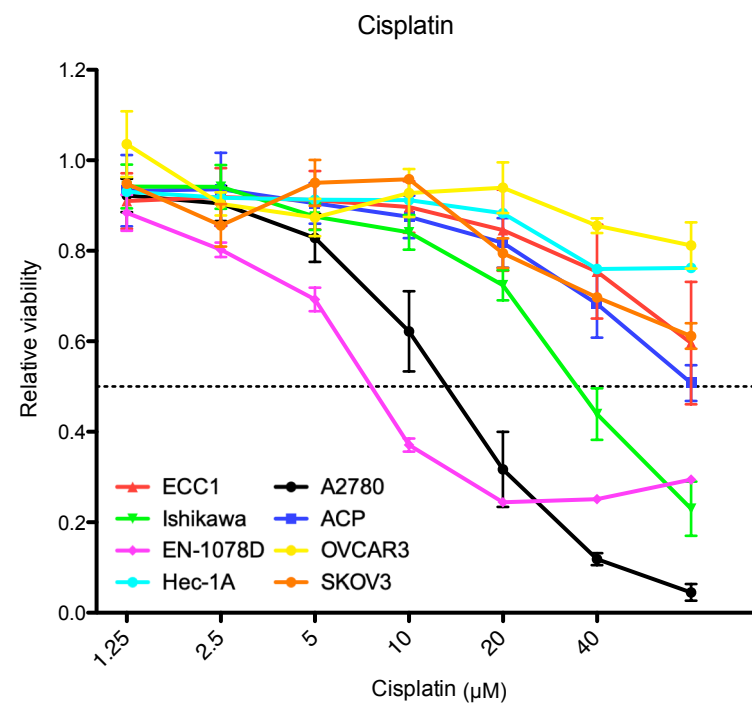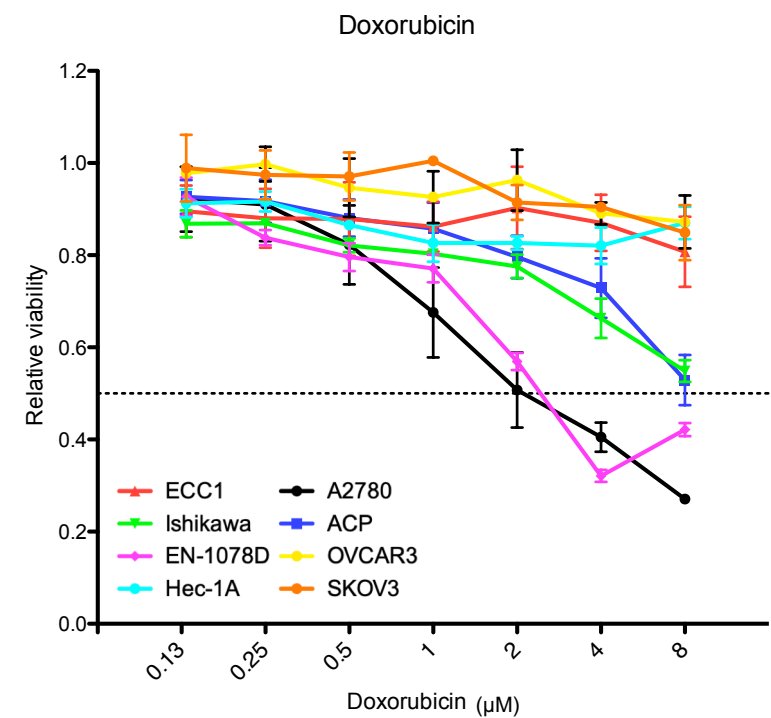

Supplement: Supplementary file 1 — Fig. S1. Effects of single agent therapy on gynecological cancer cell lines A. Cell lines were treated with increasing concentration of cisplatin (0‐80μM), doxorubicin (0‐8μM), AZD5363(0‐40μM) or NVP‐BEZ‐235(0‐4μM) for 24h. MTT assays were then used to determine changes in cell viability. All data are means ± SEM of three independent experiments. [file MOL2-15-2106-s001.pdf]
